# Antimicrobial Resistance Genes in Porcine *Pasteurella multocida* Are Not Associated with Its Antimicrobial Susceptibility Pattern

**DOI:** 10.3390/antibiotics9090614

**Published:** 2020-09-17

**Authors:** Máximo Petrocchi-Rilo, César-B. Gutiérrez-Martín, Esther Pérez-Fernández, Anna Vilaró, Lorenzo Fraile, Sonia Martínez-Martínez

**Affiliations:** 1 Departamento de Sanidad Animal, Unidad de Microbiología e Inmunología, Universidad de León, s/n, 24071 León, Spain; mpetr@unileon.es (M.P.-R.); eperf@unileon.es (E.P.-F.); smarm@unileon.es (S.M.-M.); 2 Grup de Sanejament Porcí, 25192 Lleida, Spain; micro@gsplleida.net; 3 Departament de Ciència Animal, ETSEA, Universitat de Lleida-Agrotecnio, 25198 Lleida, Spain; Lorenzo.fraile@udl.cat

**Keywords:** *Pasteurella multocida*, antimicrobial resistance genes, antimicrobial susceptibility patterns, swine

## Abstract

Forty-eight *Pasteurella multocida* isolates were recovered from porcine pneumonic lungs collected from farms in “Castilla y León” (north-western Spain) in 2017–2019. These isolates were characterized for their minimal inhibition concentrations to twelve antimicrobial agents and for the appearance of eight resistance genes: *tetA*, *tetB*, *bla_ROB1_*, *bla_TEM_*, *ermA*, *ermC*, *mphE* and *msrE*. Relevant resistance percentages were shown against tetracyclines (52.1% for doxycycline, 68.7% for oxytetracycline), sulphamethoxazole/trimethoprim (43.7%) and tiamulin (25.0%), thus suggesting that *P. multocida* isolates were mostly susceptible to amoxicillin, ceftiofur, enrofloxacin, florfenicol, marbofloxacin and macrolides. Overall, 29.2% of isolates were resistant to more than two antimicrobials. The tetracycline resistance genes (*tetA* and *tetB*) were detected in 22.9% of the isolates, but none were positive to both simultaneously; *bla_ROB1_* and *bla_TEM_* genes were found in one third of isolates but both genes were detected simultaneously in only one isolate. The *ermC* gene was observed in 41.7% of isolates, a percentage that decreased to 22.9% for *msrE*; finally, *ermA* was harbored by 16.7% and *mph*E was not found in any of them. Six clusters were established based on hierarchical clustering analysis on antimicrobial susceptibility for the twelve antimicrobials. Generally, it was unable to foresee the antimicrobial susceptibility pattern for each family and the association of each particular isolate inside the clusters established from the presence or absence of the resistance genes analyzed.

## 1. Introduction

The Porcine Respiratory Disease Complex (PRDC) is a syndrome that results from a combination of infectious and non-infectious factors. *Pasteurella multocida* is one of the most common bacterial agents isolated from respiratory clinical cases [1]. It belongs to the commensal organisms in the upper portion of the porcine respiratory tract that can also cause pneumonia in growing and finishing pigs worldwide. *P. multocida* is normally considered as a secondary agent but it has also been described as a primary agent of haemorrhagic septicaemia in pigs, mainly caused by B:2 [2] or E:5 serotypes [3]. Moreover, the prevalence of *P. multocida* serotypes can vary considerably from region to region and over time in a given region [4].

The use of antimicrobials could be necessary to control bacteria entailed in PRDC with a therapeutic or a metaphylactic goal [5], but their use may be one of the factors involved in the emergence and spread of bacterial resistance from pig origin across the world [6,7]. Although *P. multocida* had been generally susceptible to the majority of antimicrobials, the emergence of multidrug-resistant pathogenic bacteria has been widely reported in recent times probably associated with the abusive use of antimicrobials [4]. Tetracyclines have been used for prophylaxis, in such a way that the effects of long-term consumption of these drugs probably resulted in increased levels of resistance [8,9], with global problems for public health [10]. Some of these resistance genes are often located on mobile genetic elements, frequently transmissible plasmids and transposons [11]; in addition, exchanges of resistance genes are common not only in the genus *Pasteurella*, but also in the family *Pasteurellaceae* [12].

The term of antimicrobial resistome has been proposed for describing the collection of all known antimicrobial resistance genes in the microbial ecosystem [13]. This concept supports the theory that resistant organisms and their antimicrobial resistome are settled after birth in living beings and are gained from the mother or by direct contact with resistant bacteria in the adjoining environment [14].

In this study, the antimicrobial susceptibility patterns observed in *P. multocida* strains isolated from pigs in Spain between 2017 and 2019 was linked with the presence or absence of antimicrobial resistance genes in order to decipher whether it is possible to determine the feasibility of selecting antimicrobials from the identification of resistance genes by molecular biology.

## 2. Results

### 2.1. Antimicrobial Resistance

The MIC (minimum inhibitory concentration) range, MIC_50_, MIC_90_ and antimicrobial resistance of the 48 *P. multocida* isolated from porcine pneumonic lungs in Spain from 2017 to 2019 are shown in Table 1. All isolates were susceptible to ceftiofur, florfenicol, tildipirosin and tulathromycin, while most of them (>95%) were susceptible to amoxicillin, the two quinolones tested (enrofloxacin and marbofloxacin), and tilmicosin. In addition, 25% of isolates showed resistance to tiamulin and 31.2% or 43.7% to sulphamethoxazole/trimethoprim depending on the selected breakpoint (*Staphylococcus aureus* and *Escherichia coli*, or *Streptococcus suis*, respectively). On the other hand, doxycycline and oxytetracycline cannot be used to treat 52.1% and 68.7% of the cases, respectively. In addition, the distribution of the MIC range of amoxicillin, doxycycline, tiamulin, tilmicosin and tulathromycin was clearly unimodal, whereas *P. multocida* isolates seemed to show a bimodal distribution to enrofloxacin, and a multimodal bend to sulphamethoxazole/trimethoprim. Tailing of isolates over the MIC range was found for ceftiofur, marbofloxacin, oxytetracycline and tildipirosin (Table 1).

Overall, 89.6% of the isolates (*n* = 43) were resistant to one or more antimicrobial agents, in such a way that 25.0% (*n* = 12) showed resistance to only one compound; 35.4% (*n* = 17) to two antimicrobial agents; 22.9% (*n* = 11) to three drugs and 6.2% (*n* = 3) to four antimicrobials simultaneously. The most common resistance pattern was observed for the two tetracyclines tested, with 12 isolates being resistant to both of them. On the other hand, only 10.4% (*n* = 5) of the isolates were susceptible to all 12 compounds evaluated (Table 2).

### 2.2. Description of Resistance Genes

Of the eight resistance genes examined, *tetB* was harbored by 39.6% of *P. multocida* isolates, while *tetA* was only borne by 12.5%. Globally, 22.9% of them showed one of the two tetracycline resistance genes, but none was positive to both simultaneously. With regard to β-lactam resistance genes, 27.1% of isolates were positive to *bla_ROB1_*, while only 8.3% were to *bla_TEM_*, in such a way that one third of isolates showed resistance to some of these two genes, and only one carried both *bla_ROB1_* and *bla_TEM_* genes. In addition, 41.7% of isolates showed the *ermC* gene, a figure that decreased until 22.9% to *msrE*; *ermA* was harboured by 16.7% and, finally, *mphE* was not found in any isolate. A total of 27.1% of isolates amplified one of the macrolide resistance genes; the same percentage amplified two of them, and 2.1% amplified three macrolide resistance genes at the same time.

### 2.3. Analysis of the Association between the Presence of Resistance Genes and Antimicrobial Patterns

Only in 19 cases (8.3% for the *tetA* gene, 29.2% for the *tetB* gene and 2.1% for the *bla_ROB1_* gene) could a clear association be established between the resistance to a given antimicrobial agent and the detection of some of the genes being able to explain this lack of susceptibility (Table 3). Interestingly, this association was observed for tetracyclines in 18 of them (94.7%). On the contrary, the existence of 19 isolates carrying the *ermC* gene but being susceptible to the three macrolides evaluated, or the 15 isolates with the *bla_ROB1_* gene but without resistance to amoxicillin must be highlighted (Table 3). Globally, the identification of resistance genes in 62 cases could not be associated with the susceptibility pattern observed for tetracyclines, β-lactams or macrolides (Table 3). Thus, no significant association between the presence of resistance genes and that of a resistant phenotype for one particular antimicrobial agent was observed (Table 4).

### 2.4. Relationship between the Presence of Resistance Genes and Clusters based on Antimicrobial Susceptibility Pattern of 12 Antimicrobials

**Figure 1:**
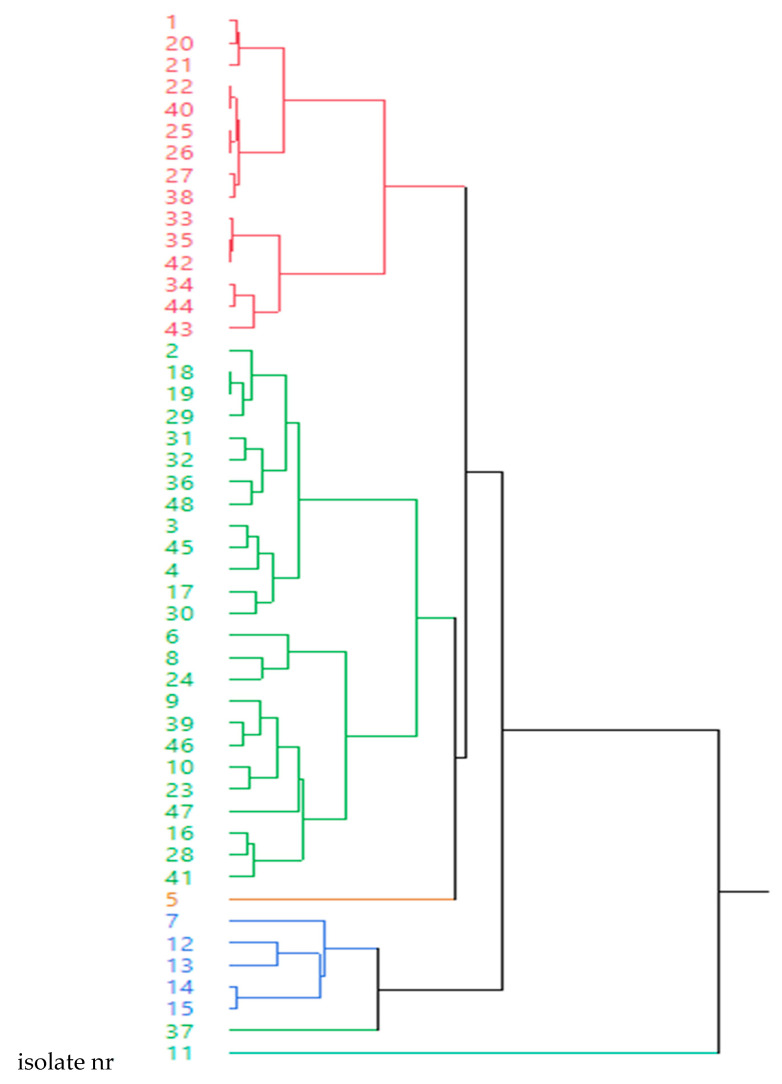
Dendrogram showing the results of 48 *Pasteurella multocida* isolates after a hierarchical clustering analysis of MIC values for the 12 antimicrobials tested.

*P. multocida* isolates were grouped into six clusters (Figure 1) and MIC values of these 48 isolates after a hierarchical clustering analysis for the 12 antimicrobial agents tested are shown in Table 5. Thus, cluster 1 shows low MIC values for most antimicrobials except for sulphamethoxazole (4 μg/mL) and oxytetracycline in six isolates. Cluster 2 shows low MIC values for all the antimicrobial families with the exception of pleuromutilins for most strains. Cluster 3 is similar to cluster 2 but MICs for amoxicillin and oxytetracycline were extremely high (8 μg/mL) and MICs for pleuromutilins were close to MIC_50_ for this isolate. Cluster 4 shows low MIC values for all antimicrobial families with the exception of quinolones for most strains. Cluster 5, which contains only one isolate, is similar to cluster 4, but the MIC values for tetracyclines and pleuromutilins were also high for this isolate. Finally, cluster 6 (one isolate) shows a peculiar susceptibility pattern with very high MICs for macrolides (64 μg/mL for tildipirosin, tilmicosin and tulathromycin), quinolones and tetracyclines (Figure 1 and Table 5). The presence of *tetA* and *ermA* genes was significantly associated with clusters 2 and 5 (*p* = 0.048) and clusters 2, 4 and 6 (*p* < 0.0001), respectively. For the remaining genes, no significant association with any of the clusters was seen.

## 3. Discussion

Spain is one of the European countries with a higher antibiotic consumption in animals (2,964 tonnes of active substance in 2014) [15], and this fact must be taken into account in studies addressing the resistance percentages for antimicrobial agents in pathogenic bacteria. One of the critical points is the selection of antimicrobials to be tested in vitro for further use in swine production; in this study, the most frequently used antimicrobials for treating respiratory diseases in pigs were compared. Surprisingly, only one *P. multocida* isolate among the 48 tested was found to be resistant to amoxicillin in our investigation, opposite to the 13/32 resistant isolates (40.6%) reported also in Spain one year ago to ampicillin [16], a very similar β-lactam antibiotic. The resistance to this group of compounds has been linked mainly with the presence of the *bla_ROB1_* resistance gene, not only in *P. multocida* [17] but alo in other genera and species of *Pasteurellaceae*, such as *Actinobacillus pleuropneumoniae* [18] or *Glässerella parasuis* [19]. In fact, the isolate resistant to amoxicillin harbored this resistance gene. On the other hand, eleven isolates showing the *bla_ROB-1_* gene, three bearing the *bla_TEM_* gene and even another one sharing both genes were susceptible to amoxicillin; consequently, these genes were present but were not expressed in these isolates. Just as in our study, a lower appearance of *bla_TEM_* compared to *bla_ROB1_* has been previously observed [16,20]. A similar behavior has been reported in Spain for 30 years for ceftiofur, a broad-spectrum third-generation cephalosporin [16,21] which was approved for treatment of swine respiratory tract diseases approximately at that time. Its resistance has been linked to the *bla_TEM_* gene [22]. Even though this gene has been detected in four *P. mutocida* isolates, all of them have shown susceptibility to ceftiofur.

The resistance rates for tetracyclines in this study were almost four times higher (for oxytetracycline) or almost three times higher (for doxycycline) than those reported only one year before also in Spain; however, detection of the *tetB* gene was similar in both investigations [19]. This one has been most frequently found the *tet* gene [19,23], not only in *P. multocida* but also in other *Pasteurellaceae*, such as *A. pleuropneumoniae* [9]. The presence of *tetB* gene suggests that the mechanism underlying the resistance to tetracyclines involves efflux pump proteins that move these compounds out of the bacteria, so causing the inactivity of tetracyclines against *P. multocida* [24]. The spread of this gene has been related with either its presence in transmissible plasmids and transposons, such as pB1001 and Tn*10*, respectively [11], or to clonal dissemination rather than horizontal transfer of plasmids [23].

As in a previous study [19], enrofloxacin and marbofloxacin behaved as two of the highest in vitro effective antibiotics against the isolates. Even so, one of the clinical strains (2.1%) was resistant to enrofloxacin, a percentage much smaller than the 22.5% found for this fluoroquinolone by Oh et al. in Brazil [23]. Florfenicol is a safe phenicol used exclusively for the treatment of pneumonias caused by *P. multocida*; in this way, it was completely active against these 48 isolates. Tiamulin is an antibiotic used in the treatment of several infections in swine. Although this compound was proposed as a proper antibiotic against animal *Pasteurella* spp. [20], the 25% level of resistance observed in this investigation, albeit lower than that reported two decades ago [21], does not advise its use against pneumonias caused by *P. multocida*.

Macrolides showed excellent effective results, with only one isolate (2.1%) being resistant to tilmicosin but not harboring any of the three macrolide resistance genes studied. Quite similar resistance rates were found in Spain for the last 30 years [21]; however, a substantially higher inefficacy (12.5%) was recently demonstrated for erythromycin [16].

Fourteen resistance *P. multocida* panels were obtained in this study (29.2% over 48 isolates), with a spread lower than that seen fifteen years ago (38.5%) [25], and especially lower than the 56.2% reported in the last five years [16]. Although the rate of isolates behaving as resistant to at least two of the antimicrobial agents here compared were almost 20 points below the rate reported in 2018 (84.4% vs. 64.5%) [16]; these results suggest the need for a restrictive use of antimicrobial agents in porcine husbandry, especially that of tetracyclines, sulphametoxozole/trimethoprim and tiamulin. Other investigators [26] showed 36.6% of *P. multocida* isolates being multirresistant in Brazil, a percentage considerably lower than that seen in this study. The multiresistance in *P. multocida* to tetracyclines and sulfonamides has been previously related, not with large plasmids as in most Gram-negative organisms, but with small plasmids of 4–6 kb in size [17].

On the basis of these results, the identification of the eight antimicrobial resistance genes does not enable us to foresee the behavior of the 48 *P. multocida* isolates to amoxicillin, doxycycline, oxytetracycline, tildiporison, tilmicosin and tulathromycin, as there is absence of a significant association between both parameters. To our knowledge, this is the first investigation in which such a noticeable mismatch between phenotypic and genetic characterization of resistances in *P. multocida* is reported. Similarly, after grouping isolates into six clusters according to their antimicrobial sensitivity behavior, only an association among these clusters and the presence or not of resistance genes could be established for the *tetA* and *ermA* genes. Nevertheless, this association was not linked to the antimicrobial susceptibility pattern described for each cluster. Thus, the presence of the *tetA* gene was significantly associated with clusters 2 and 5, and showed a very different pattern and it was not associated with resistance to tetracyclines in the case of cluster 2 for most isolates. Cluster 5 contained only one isolate and, therefore, this result must be assessed with caution. In the case of the *ermA* genes, its presence was significantly associated with clusters 2, 4 and 6 that had very different antimicrobial susceptibility patterns. Curiously, cluster 6 showed high MIC values for macrolides, and the *ermA* gene was present. In short, the presence of resistance genes cannot be associated with antimicrobial susceptibility for all the families tested. Therefore, these results clearly recommend carrying out phenotypic characterization in order to optimize the use of antimicrobials under field conditions. This point is critical taking into account a one-health approach in connection with the use of antimicrobials in livestock.

## 4. Material and Methods

### 4.1. Clinical Samples

Clinical samples were taken between 2017 and 2019 in farms in “Castilla y León” (north-western Spain) from diseased or recently deceased pigs with acute clinical signs of respiratory tract infections that had not been exposed to antimicrobial treatment for at least 15 days prior to sampling. Thus, the pigs included in the sampling procedure were three to 24 weeks old, with overt clinical signs such as loss of appetite, apathy, hyperthermia (>39.8 °C), and significantly increased mortality rates vs. baseline situation due mainly to respiratory disorders in intensive farms. In each case, at least two animals with these clinical signs were humanely sacrificed, and lung samples of these animals or from recently deceased pigs (<12 h) were drawn.

All experimental procedures were approved by the Ethics Committee for Animal Experimentation of the University of Lleida and performed in accordance with authorization 10343 issued by the Catalan Department of Agriculture, Livestock, Fisheries and Food (Section of biodiversity and hunting).

### 4.2. Bacterial Isolation and Identification

Clinical specimens were grown aseptically on Columbia blood agar base supplemented with 5% of defibrinated sheep blood (Oxoid), chocolate blood agar (GC II agar with IsoVitaleX, BD) and MacConkey agar (Biolife). All plates were incubated at 35–37 °C in aerobic conditions with 5–10% CO_2_ for 24–48 h. Identification of isolates was carried out by matrix assisted laser desorption ionization-time of flight (MALDI-TOF) mass espectrometry (Biotyper System, Bruker Daltonics, Bremen, Germany) as previously described [24].

### 4.3. Antimicrobial Sensitivity Testing

Bacteria were cultured on Columbia blood agar and incubated at 35–37 °C in ambient air (or with 5–10% CO_2_) for 18–24 h. MICs were determined using the broth microdilution method by means of customizing 96-well microtiter plates (Sensititre, Trek Diagnostic Systems Inc., East Grinstead, UK) containing 12/7 or 8 antimicrobials/concentrations, respectively, in accordance with the recommendations presented by the Clinical and Laboratory Standards Institut CLSI [16,17]. The antimicrobial agents tested were amoxicillin, ceftiofur, doxycycline, enrofloxacin, florfenicol, marbofloxacin, sulphamethoxazole/trimethoprim, oxytetracycline, tiamulin, tilmicosin, tildipirosin and tulathromycin. This panel was selected in order to represent the commonly used compounds for treatment of pig respiratory diseases in farms. Three to five colonies were picked and emulsified in demineralized water to obtain a turbidity of 0.5 McFarland standard (Sensititre™ nephelometer V3011). Suspensions were further diluted in cation-adjusted Mueller-Hinton broth to reach a final inoculum concentration of 5 × 10^5^ CFU/mL. Then, the panel was reconstituted by adding 100 μL/well of the inoculum, and plates were incubated at 35 ± 2 °C for 18–24 h [27,28]. The antibiotic panels were read manually using Sensititre™ Vizion (V2021) and the MIC value was established as the lowest concentration inhibiting visible growth. A colony count and a purity check were performed for each clinical strain following CLSI and manufacturer recommendations. Moreover, control *P. multocida* strains were also included in all the susceptibility testing runs as quality control [27,28]. The MICs of the quality control strains had to be within acceptable CLSI ranges to authenticate the results obtained in the laboratory.

### 4.4. Determination of Antimicrobial Resistance Genes

Eight antibiotic resistance genes, corresponding to three antimicrobial families, were tested: tetracyclines (*tetA*, *tetB*), β-lactams (*bla_ROB1_*, *bla_TEM_*) and macrolides (*ermA*, *ermC*, *msrE*, *mphE*). The primers used are shown in Table 6. The PCRs were performed in an Eppendorf Mastercycler^®^ thermocycler by using 0.2 mL tubes containing 47 μL of PCR master mix and 3 μL of DNA sample (primers used and annealing temperatures are shown in Table 6). A volume of 10 μL of each reaction mixture was analyzed by electrophoresis in an agarose gel. The PCR products were stained with RedSafe™ and visualized under UV light.

### 4.5. Data Analysis

A strain was considered susceptible to one antimicrobial agent if its MIC value was below its clinical breakpoint. Clinical breakpoints from the CLSI were used when available [16,17] and they were extrapolated from clinical breakpoints of other organisms when data from the CLSI were not available (Table 1). Moreover, MIC distributions were used to define MIC_50_, MIC_90_, being determined respectively as the MICs inhibiting 50% and 90% of isolates. 

### 4.6. Statistical Analysis

SPSS software version 2.1 was used to carry out the statistical analysis. In all the cases, *p*-values ≤0.05 were considered significant. A multivariate analysis was applied on the MIC of the 12 antimicrobials for all the strains. Thus, a dendrogram was generated using between-group linkage via Ward’s hierarchical clustering that allows generating clusters of strains according to their antimicrobial susceptibility testing for all the antimicrobials together. A chi-square test was used to determine the association between the isolates harboring or not a resistance gene to a certain antimicrobial family and its association with the clusters determined based on hierarchical clustering analysis.

## 5. Conclusions

Ceftiofur, florfenicol, tildipirosin and tulathromycin were highly effective in vitro against the *P. multocida* isolates tested and, therefore, they remain suitable for the treatment of porcine respiratory infections due to this pathogen. However, the identification of β-lactam, tetracycline and macrolide resistance genes did not allow the prediction of antimicrobial resistances for these families. For this reason, knowledge of the antimicrobial susceptibility patterns (MICs) becomes essential to implement a prudent use of antimicrobials under field conditions.

## Figures and Tables

**Table 1:** MIC (minimum inhibitory concentration) range, MIC_50_, MIC_90_ and percentage of resistant *Pasteurella multocida* isolates recovered in Spain between 2017 and 2019.

Antimicrobial Agent	Range (μg/mL)	MIC_50_ (μg/mL)	MIC_90_ (μg/mL)	Breakpoint (μg/mL) *	Antimicrobial Resistance (%)
Amoxicillin	1–8	0.25	8	0.5 ^$^	2.1
Ceftiofur	0.06–0.25	0.06	0.12	2	0
Doxycycline	0.25–2	1	>2	0.5 ^$$^	52.1
Enrofloxacin	0.03–0.5	0.03	0.12	0.25	2.1
Florfenicol	0.5	0.5	0.5	2	0
Marbofloxacin	0.03–0.5	0.03	0.12	0.25 ^&^	4.2
Oxytetracycline	0.5–8	2	>8	0.5	68.7
Sulphamethoxazole/trimethoprim (19/1 ratio) ^§^	0.06–4	0.25	>4	0.5 ^&&^ 2 ^§§^	43.7 31.2
Tiamulin	2–32	16	>32	16	25
Tildipirosin	0.5–4	1	4	4	0
Tilmicosin	2–32	8	32	16	2.1
Tulathromycin	0.5–4	1	2	16	0

* Clinical breakpoints were obtained from CLSI VET08 or CLSI M100 with the following clarifications: ^$^ extrapolated from ampicillin. ^$$^ Extrapolated from tetracycline. ^&^ Extrapolated from enrofloxacin. ^&&^ Extrapolated from *Streptococcus suis.* ^§^ MIC is for trimethoprim in this table. ^§§^ Extrapolated from *Staphylococus hyicus* and *Escherichia coli*.

**Table 2:** Antimicrobial resistance profiles of 48 *Pasteurella multocida* strains in this study.

Number of Isolate	Number of Antimicrobial Agents	Resistance to
5	0	No antimicrobial resistance
2	1	Oxytetracycline
6	1	Sulphamethoxazole/trimethoprim
4	1	Tiamulin
12	2	Doxycycline + oxytetracycline
1	2	Marbofloxacin + oxytetracycline
3	2	Oxytetracycline + sulphamethoxazole/trimethoprim
1	2	Oxytetracycline + tiamulin
1	3	Amoxicillin + doxycycline + oxytetracycline
4	3	Doxcycline + oxytetracycline + sulphamethoxazole/trimethoprim
5	3	Doxcycline + oxytetracycline + tiamulin
1	3	Oxytetracycline + tiamulin + tilmicosin
1	4	Doxycycline + enrofloxacin + oxytetracycline + tiamulin
2	4	Doxycycline + oxytetracycline + sulphamethoxazole/trimethoprim + tiamulin

**Table 3:** Association between the presence of resistance genes and antimicrobial susceptibility patterns in 48 *Pasteurella multocida* isolates.

Resistance Gene	Number of Isolates	Resistance or Sensitivity	Resistance or Sensitivity to
*tetA*	3	Resistance	Tetracyclines *
*tetA*	1		Oxytetracycline
*tetB*	11		Tetracyclines *
*tetB*	3		Oxytetracycline
*bla_ROB1_*	1		Amoxicillin
*tetA*	2	Sensitivity	Tetracyclines *
*tetB*	5		Tetracyclines *
*bla_ROB1_*	15		Amoxicillin
*ermA*	8		Macrolides ^$^
*ermC*	19		Macrolides ^$^
*msrE*	12		Macrolides ^$^
*mphE*	1		Macrolides ^$^

* Tetracyclines are doxycycline and oxytetracycline. ^$^ Macrolides are tildipirosin, tilmicosin and tulathromycin.

**Table 4:** *p*-values obtained after studying the association between resistance genes and a phenotype resistant for β -lactams, macrolides and tetracyclines in the 48 *Pasteurella multocida* isolates.

Antimicrobial Resistance Genes *		β-lactams	Macrolides ^$^	Tetracyclines	
		Amoxicillin	Tilmicosin	Doxycycline	Oxitetracycline
β-lactam resistance genes	*bla_ROB1_*	0.5536	-	-	-
	*bla_TEM_*	0.8408	-	-	-
Macrolide resistance genes	*ermA*	-	0.7764	-	-
	*ermC*	-	0.6538	-	-
	*msrE*	-	0.7392	-	-
Tetracycline resistance genes	*tetA*	-	-	0.9131	0.9063
	*tetB*	-	-	0.5146	0.7255

* Only resistance genes to three antibiotic families were tested (β-lactams, macrolides and tetracyclines). ^$^ Tilmicosin was the only macrolide tested because no resistant strains were obtained for tildipirosin and tulathromycin.

**Table 5:** MIC values of the 48 *Pasteurella multocida* isolates grouped into six clusters after a hierarchical clustering analysis for the 12 antimicrobials tested.

Cluster	Isolate nr	MIC											
		Flor	Enrof	Amox	Marb	Ceft	Sulf	Tild	Dox	Oxitet	Tia	Tulat	Tilm
1	38	0.5	0.03	0.25	0.03	0.06	4	0.5	0.25	0.5	16	1	2
	27	0.5	0.03	0.25	0.03	0.06	4	0.5	0.5	0.5	16	1	2
	22	0.5	0.03	0.25	0.03	0.06	4	0.5	0.5	0.5	16	1	4
	40	0.5	0.03	0.25	0.03	0.06	4	0.5	0.5	0.5	16	1	4
	25	0.5	0.03	0.25	0.03	0.06	4	0.5	0.5	1	16	1	4
	26	0.5	0.03	0.25	0.03	0.06	4	0.5	0.5	1	16	1	4
	34	0.5	0.03	0.25	0.03	0.06	4	0.5	1	8	16	1	2
	44	0.5	0.03	0.25	0.03	0.06	4	0.5	1	8	16	1	4
	21	0.5	0.03	0.25	0.03	0.12	4	0.5	0.25	0.5	16	1	4
	20	0.5	0.03	0.25	0.03	0.12	4	0.5	0.5	0.5	16	1	2
	35	0.5	0.03	0.25	0.03	0.12	4	0.5	1	8	16	1	2
	42	0.5	0.03	0.25	0.03	0.12	4	0.5	1	8	16	1	2
	33	0.5	0.03	0.25	0.03	0.12	4	1	1	8	16	1	2
	43	0.5	0.03	0.5	0.03	0.06	4	0.5	2	8	16	1	4
	1	0.5	0.03	0.5	0.03	0.12	4	0.5	0.5	1	16	1	2
2	17	0.5	0.03	0.25	0.03	0.06	0.06	0.5	1	2	2	1	2
	45	0.5	0.03	0.25	0.03	0.06	0.06	0.5	2	2	8	0.5	4
	3	0.5	0.03	0.25	0.03	0.06	0.06	1	2	4	8	1	8
	36	0.5	0.03	0.25	0.03	0.06	0.25	0.5	0.25	0.5	16	1	4
	29	0.5	0.03	0.25	0.03	0.06	0.06	1	2	2	16	1	8
	18	0.5	0.03	0.25	0.03	0.06	1	1	2	2	16	1	8
	19	0.5	0.03	0.25	0.03	0.06	1	1	2	2	16	1	8
	2	0.5	0.03	0.5	0.03	0.06	0.06	1	2	4	16	2	8
	31	0.5	0.03	0.25	0.03	0.06	0.25	2	1	2	16	2	16
	32	0.5	0.03	0.25	0.03	0.06	0.06	2	0.5	0.5	16	4	16
	46	0.5	0.03	0.25	0.03	0.06	1	1	0.25	0.5	32	1	8
	8	0.5	0.03	0.25	0.03	0.06	2	1	1	8	32	1	16
	41	0.5	0.03	0.25	0.03	0.06	0.06	2	2	2	32	2	16
	24	0.5	0.03	0.25	0.03	0.06	0.06	2	2	8	32	2	16
	28	0.5	0.03	0.25	0.03	0.06	0.25	2	2	2	32	2	8
	16	0.5	0.03	0.25	0.03	0.06	1	2	2	4	32	2	8
	48	0.5	0.03	0.25	0.06	0.06	0.06	1	1	2	16	2	8
	4	0.5	0.03	0.5	0.03	0.06	0.06	2	2	2	8	2	16
	6	0.5	0.03	0.5	0.03	0.06	0.06	2	1	8	16	2	8
	39	0.5	0.03	0,5	0.03	0.06	0.12	2	0.5	0.5	32	2	8
	47	0.5	0.03	0,5	0.03	0.06	0.06	4	0.5	1	32	4	32
	30	0.5	0.06	0.12	0.12	0.06	0.06	1	1	2	8	1	4
	10	0.5	0.06	0.25	0.06	0.12	0.5	2	0.5	0.5	32	4	8
	9	0.5	0.06	0.25	0.12	0.06	1	2	0.25	0.5	32	4	16
	23	0.5	0.12	0.25	0.12	0.12	0.06	1	0.5	1	32	2	8
3	5	0.5	0.03	8	0.03	0.06	0.25	2	1	8	16	4	16
4	7	0.5	0,03	0.5	0.03	0.25	0.12	0.5	0.5	1	16	2	4
	14	0.5	0.25	0.25	0.25	0.12	0.12	0.5	0.5	0.5	16	1	4
	12	0.5	0.25	0.25	0.25	0.25	0.25	1	0.5	0.5	16	2	8
	15	0.5	0.25	0.5	0.25	0.12	0.12	0.5	0.5	1	16	2	4
	13	0.5	0.25	0.5	0.5	0.25	0.12	0.5	0.5	1	16	2	4
5	37	0.5	0.5	0.25	0.5	0.06	0.06	1	2	4	32	2	8
6	11	2	0.5	0.25	0.5	0.12	0.25	64	8	8	16	64	64

Flor: florfenicol; enrof: enrofloxacin; amox: amoxicillin; marb: marbofloxacin; ceft: ceftiofur; tild: tildipirosin; dox: doxycycline; oxitet: oxitetracycline; tia: tiamulin; tulat: tulathromycin; tilm: tilmicosin.

**Table 6:** Pimers used in the PCRs carried out for the detection of eight antimicrobial resistance genes in 48 *Pasteurella multocida* isolates.

Resistance Gene	Primer	Amplicon Size	Annealing Temperature	Reference
*tetA*	**F**: 5′-GTA ATT CTG AGC ACT GTC GC-3′	1057 pb	62 °C	[20]
	**R**: 5′-CTG CCT GGA CAA CAT TGT TT-3′			
*tetB*	**F**: 5′CCT TAT CAT GCC AGT CTT GC-3′	774 pb	50 °C	[20]
	**R**: 5′ ACT GCC GTT TTT TTC GCC-3′			
*bla_ROB1_*	**F**: 5′ CAT TAA CGG CTT GTT CGC-3′	852 pb	55 °C	[20]
	**R**: 5′-CTT GCT TTG CTG CAT CTT-3′			
*bla_TEM_*	**F**: 5′GAG TAT TCA ACA TTT TCG T-3′	856 pb	55 °C	[20]
	**R**: 5′-ACC AAT GCT TAA TCA GTG A-3′			
*ermA*	**F**: 5′-ACG ATA TTC ACG GTT TAC CCA CTT-A-3′	610 pb	53 °C	[20]
	**R**: 5-AAC CAG AAA AAC CCT AAA GAC ACG-3′			
*ermC*	**F**: 5′-AAT-CGG CTC AGG AAA AGG-3′	562 pb	55 °C	[20]
	**R**: 5′-ATC GTC ATT TCC TGC ATG-3′			
*msrE*	**F**: 5′-TAT AGC GAC TTT AGC GCC AA-3′	271 pb	58 °C	[20]
	**R**: 3′-GCC GTA GAA TAT GAG CTG AT-3′			
*mphE*	**F**: 5′-ATG CCC AGC ATA TAA ATC GC-3′	295 pb	58 °C	[20]
	**R**: 5′-ATA TGG ACA AAG ATAGCC CG-3′			
